# Preparation of Porous Scaffold Based on Poly(3-hydroxybutyrate-co-3-hydroxyvalerate-co-3-hydroxyhexanoate) and FucoPol

**DOI:** 10.3390/polym15132945

**Published:** 2023-07-04

**Authors:** João Ricardo Pereira, Ana Margarida Rafael, Asiyah Esmail, Maria Morais, Mariana Matos, Ana Carolina Marques, Maria A. M. Reis, Filomena Freitas

**Affiliations:** 1 UCIBIO—Applied Molecular Biosciences Unit, Department of Chemistry, NOVA School of Science and Technology, NOVA University Lisbon, 2819-516 Caparica, Portugal; jra.pereira@campus.fct.unl.pt (J.R.P.); am.rafael@campus.fct.unl.pt (A.M.R.); a.esmail@campus.fct.unl.pt (A.E.); m.matos@campus.fct.unl.pt (M.M.); amr@fct.unl.pt (M.A.M.R.); 2 Associate Laboratory i4HB-Institute for Health and Bioeconomy, NOVA School of Science and Technology, NOVA University Lisbon, 2819-516 Caparica, Portugal; 3 CENIMAT/i3N, Materials Science Department, NOVA School of Science and Technology, NOVA University Lisbon, 2819-516 Caparica, Portugal; md.morais@campus.fct.unl.pt (M.M.); accm@campus.fct.unl.pt (A.C.M.)

**Keywords:** poly(3-hydroxybutyrate-co-3-hydroxyvalerate-co-3-hydroxyhexanoate) (PHBHVHHx), exopolysaccharide (EPS), FucoPol, porous scaffold, emulsion templating

## Abstract

This work focused on the development of porous scaffolds based on biocomposites comprising two biodegradable and biocompatible biopolymers: a terpolyester, poly(3-hydroxybutyrate-co-3-hydroxyvalerate-co-3-hydroxyhexanoate) (PHBHVHHx), and the bacterial polysaccharide FucoPol. The PHBHVHHx terpolymer was composed of 3-hydroxybutyrate (55 wt%), 3-hydroxyvalerate (21 wt%), and 3-hydroxyhexanoate (24 wt%). This hydrophobic polyester has low crystallinity and can form elastic and flexible films. Fucopol is a fucose-containing water-soluble polysaccharide that forms viscous solutions with shear thinning behavior and has demonstrated emulsion-forming and stabilizing capacity and wound healing ability. Emulsion-templating was used to fabricate PHA-based porous structures in which FucoPol acted as a bioemulsifier. Compared with the scaffolds obtained from emulsions with only water, the use of FucoPol aqueous solutions resulted in structures with improved mechanical properties, namely higher tensile strength (4.4 MPa) and a higher Young’s Modulus (85 MPa), together with an elongation at break of 52%. These features, together with the scaffolds’ high porosity and pore interconnectivity, suggest their potential to sustain cell adhesion and proliferation, which is further supported by FucoPol’s demonstrated wound healing ability. Therefore, the developed PHBHVHHx:FucoPol scaffolds arise as innovative porous bioactive structures with great potential for use in tissue engineering applications.

## 1. Introduction

Emulsion-templating is a promising approach for the fabrication of scaffolds with high porosity (up to 99%) and interconnected pores [1], which are requirements for a variety of applications, including tissue engineering [2,3]. This technique comprises the preparation of an emulsion with two phases (one organic and one aqueous), where the aqueous phase is dispersed in the organic solution, and when the organic solvent evaporates, it exposes the solid biopolymer that was previously dissolved, creating a structured material. The droplets of the aqueous phase will act as templates to produce highly porous scaffolds upon evaporation. This method not only produces structures with high porosity and interconnectivity, but it also allows for the adjustment of the scaffolds’ porosity by changing the aqueous phase volume [1,4]. This method has been utilized for the preparation of porous scaffolds based on different polymers, including polyhydroxyalkanoates (PHAs). Examples include the porous scaffolds based on the co-polymer poly(3-hydroxybutyrate-co-3-hydroxyvalerate) (PHBHV), developed by Ruiz (2011) [5], characterized by having some interconnected porosity throughout the structure, together with an average pore size of 7 µm. Esmail (2021) was also able to produce porous scaffolds with the emulsion templating method using PHBHV and the homopolymer poly(3-hydroxybutyrate) (PHB) [6]. The resulting porous structures had interconnected pores (porosity between 27 and 49%) and a tensile strength at break of 3.35 MPa and 3.18 MPa, respectively. Bergstrand (2012) fabricated porous PHB scaffolds with a porosity of approximately 52% [7].

Poly(3-hydroxybutyrate-co-3-hydroxyvalerate-co-3-hydroxyhexanoate) (PHBHVHHx) is considered one of the most promising additions to the PHAs family because it combines short-chain length (scl) and medium-chain length (mcl) monomers into terpolyesters with interesting novel properties [8,9]. When compared with either scl- or mcl-PHAs, PHBHVHHx seems to combine the tensile strength properties of the PHB homopolymer (5.0–15.7 MPa) with the flexibility of mcl-PHA polymers (elongation in the range of 133–340%) [10,11]. Adding to the improvement of the mechanical properties, PHBHVHHx also demonstrated higher biocompatibility and potential for cell culture than other materials used for biomedical applications, including poly(lactic acid) (PLA), PHBHV, and poly(3-hydroxybutyrate-co-3-hydroxyhexanoate) (PHBHHx) [12,13].

Emulsion systems are inherently unstable, often requiring the addition of surfactants to improve their stability [14]. Usually the surfactants used in emulsion-templating techniques are either synthetic terpolymers (such as polyethyleneoxide (PEO) and polypropyleneoxide (PPO)) or high hydrophile-lipophile molecules (for instance, Triton X-405 and Span 80 (sorbitan monooleate)) [15]. However, for tissue engineering applications, the presence of surfactants on the material can be a major issue due to their non-biodegradability, possible inherent toxicity, and the possibility that they could induce allergic reactions when in contact with human skin [16], a step that can make the technique quite laborious and expensive, together with the surfactants’ cost [1,4]. Therefore, the utilization of natural biodegradable and biocompatible surfactants, such as polysaccharides, could be a major step towards the development of new materials for tissue engineering applications. Dextran has already been studied as an emulsifier agent in water-in-chloroform emulsions [17], although the emulsions could not maintain their droplet size and stability for a long time. Another example was the use of arabic and xanthan gums for stabilization of chloroform-in-water emulsions; however, the produced emulsions lacked physico-chemical stability [18]. More recently, López-Ortega (2020) demonstrated the emulsifying ability of a novel exopolysaccharide (EPS) produced by *Haloferax mucosum* (DSM 27191) to stabilize emulsions between water and nonpolar solvents such as chloroform and n-hexane [19].

FucoPol is a fucose-rich EPS secreted by the bacterium *Enterobacter* A47. This biopolymer has a high molecular mass (4.19 × 10^6^–5.8 × 10^6^ Da), and it is composed of fucose, galactose, glucose, and glucuronic acid [20]. Among its various properties, FucoPol has demonstrated film-forming capacity and emulsifying ability [21,22]. Moreover, FucoPol was shown to be non-cytotoxic towards various cell lines, including human skin keratinocytes and mouse fibroblasts [20,23]. Additionally, Concórdio-Reis (2020) reported FucoPol’s ability to promote the in vitro migration of keratinocytes, suggesting its use for skin regeneration applications [23].

This study aimed to develop porous scaffolds based on two natural polymers using the emulsion-templating technique. The inherent biocompatibility of both biopolymers (PHBHVHHx and FucoPol) makes them useful materials for the development of porous scaffolds for biomedical applications (i.e., tissue engineering). This feature is important to ensure that the produced structures have reduced cytotoxicity and do not induce allergic reactions when in contact with skin. The terpolyester PHBHVHHx’s enhanced mechanical properties, together with FucoPol’s bioemulsifier capacity and skin regeneration potential, could not only improve the emulsion stabilization when producing scaffolds by the emulsion templating technique, but it also provides the produced structures with a unique opportunity to be used as tissue engineering materials with enhanced properties. The biopolymeric scaffolds were characterized in terms of morphology, water uptake degree, and mechanical properties.

## 2. Materials and Methods

### 2.1. Biopolymer Production

PHBHVHHx was produced from fruit waste in a three-stage bioprocess using mixed microbial cultures (MMCs), as described by Silva (2022) [9]. The biomass containing the biopolymer was recovered from the cultivation broth by centrifugation (10,375× *g*, 15 min, at 4 °C) and lyophilization. The biopolymer was extracted from the dry biomass (10 g) by Soxhlet extraction with chloroform (250 mL) and purified by its precipitation in ice-cold ethanol (1:10, *v*/*v*), as described by Pereira (2019) [24].

FucoPol was produced by the bioreactor cultivation of *Enterobacter A47* (DSM 23139) using glycerol as the carbon source [25]. FucoPol was recovered from the cultivation broth as described by Concórdio-Reis (2020) [25]. Briefly, the broth was collected from the bioreactor, diluted with deionized water (1:10, *v*/*v*) to reduce its viscosity, and centrifuged (13,000× *g*, 45 min) for cell removal. The cell-free supernatant was submitted to a thermal treatment (70 °C, 1 h) for protein denaturation and centrifuged (13,000× *g*, 45 min) for removal of cell fragments and denatured proteins. FucoPol was purified by diafiltration with a crossflow module (Sartocon Slide Holder, Sartorius, Goettingen, Germany) using a membrane with a surface area of 100 cm^2^ and a 100 kDa molecular weight cut-off (Hydrosart ultrafiltration cassette, Sartorius, Goettingen, Germany) for removal of low molecular weight compounds. During the diafiltration process, deionized water was added to facilitate the diffusion of low-molecular-weight molecules throughout the membrane. Afterwards, the equipment was used as an ultrafiltration unit with no water addition. The attained solution was then freeze-dried to obtain the FucoPol biopolymer.

The biopolymers were kept at room temperature in closed flasks until use.

### 2.2. Biopolymer Characterization

#### 2.2.1. Composition

PHBHVHHx composition was determined by gas chromatography (GC). Samples (1.5 mg) were mixed with 2 mL benzoic acid methyl ester (Sigma-Aldrich, Darmstadt, Germany) in chloroform (Honeywell, Charlotte, NC, USA) (1 g/L) and 2 mL 20% (*v*/*v*) sulfuric acid (Honeywell) in methanol (Fisher Chemical, Hampton, VA, USA) and heated at 100 °C for 4 h. After cooling, 1 mL of deionized water was added and mixed in a vortex. After phase separation, the aqueous phase was retrieved, and another 1 mL of deionized water was added and mixed. After phase separation, the organic phase was recovered, passed through molecular sieves, filtered using PTFE syringe filters of 0.2 µm pore (Labfil, Shaoxing, China) into a vail, and analyzed by GC (TRACE 1300, Thermo Scientific, Waltham, MA, USA) with a column of 60 m, 0.53 mmID, 1 μM df, Crossbond, and Stabilwax (Restek, Bellefonte, PA, USA). The injection volume was 1.0 µL, with a running time of 32 min, constant pressure of 14.50 psi, and helium as carrier gas. The heating ramp followed a 20 °C/min rate until 100 °C, 3 °C/min until 155 °C, and again 20 °C/min until 220 °C. The standards used for this analysis were 3-hydroxybutyric acid (for 3-hydroxybutyrate, 3HB), 3-hydroxyvaleric acid (for 3-hydroxyvalerate, 3HV), and 3-hydroxyhexanoic acid (for 3-hydroxyhexanoate, 3HHx) (97%, Sigma-Aldrich, Darmstadt, Germany) with concentrations between 0.05 and 1.0 g/L. The standards were prepared with the same protocol used for sample preparation. FucoPol’s composition was determined by high-performance liquid chromatography (HPLC) using a Carbopac PA10 column (Thermo Scientific, Waltham, MA, USA, Dionex, Sunnyvale, CA, USA), equipped with an amperometric detector. Samples of FucoPol (1 g/L, 5 mL) were hydrolyzed with 0.1 mL trifluoroacetic acid (TFA) (99%, Sigma-Aldrich, Darmstadt, Germany) at 120 °C for 2 h. L-Fucose (Biosynth, Gstaad, Switzerland), D(+)-glucose anhydrous (Scharlau, Barcelona Spain), D(+)-galactose (98%, Alfa Aesar, Ward Hill, MA, USA), and D(+)-glucuronic acid (98%, Alfa Aesar, Ward Hill, MA, USA) were used as standards.

#### 2.2.2. Molecular Mass Distribution

The number and weight average molecular weights (M_n_ and M_w_, respectively) and the polydispersity index (PDI = M_n_/M_w_) of the PHBHVHHx samples were determined by a size exclusion high performance liquid chromatography (SE-HPLC) System (KNAUER Smartline, Berlin, Germany) using monodisperse polystyrene standards (370–2520,000 Da). The samples were dissolved in chloroform (concentration range: 0.3–0.4%, *w*/*v*). The samples were analyzed by SE-HPLC with a Phenomenex Phenogel Linear Liquid Chromatographic Column 300 × 7.8 mm (Phenomenex, Torrance, CA, USA), using a temperature of equilibration of 30 °C and a flow rate of 1 mL/min of chloroform as the mobile phase. Samples were stored at 4 °C before injecting 100 µL in the SE-HPLC circuit and detected in a refractive index detector (RID) Waters2414 (Waters, Milford, CT, USA). FucoPol’s M_n_, M_w_, and PDI were determined using the same SE-HPLC system (KNAUER Smartline, Berlin, Germany) and column (Phenomenex, USA), using 0.1 M LiNO_3_ as eluent at a flow rate of 0.6 mL/min. FucoPol (50 µL) solution (0.5%, *w*/*v*, in 0.1 M LiNO_3_) was injected and detected in RID Waters2414 (Waters, Milford, CT, USA). M_w_ and M_n_ were calculated using a calibration curve generated with pullulan standards (P50 to P80).

#### 2.2.3. X-ray Diffraction

The crystalline structures of PHBHVHHx and FucoPol polymers were analyzed by X-ray diffraction (XRD) using a PANalytical’s X’Pert PRO MRD diffractometer (PANalytical B.V., Almelo, The Netherlands) with a monochromatic Cu Ka radiation source (wavelength 1.540598 Å). Data were acquired in a range between 10° and 90° (2θ) with a scanning step size of 0.03° in continuous mode and operating at 45 kV with 40 mA.

The degree of crystallinity was calculated according to the following equation [26]:

Crystallinity (%) = A_cryst_/A_total_ × 100, (1)

Here, A_Cryst_ is the sum of the area under crystalline peaks, and A_Total_ is the total area under the diffractogram. The peak deconvolution was conducted using the X’Pert HighScore Plus 3.0 software (Malvern Panalytical, Malvern, UK). The area under the deconvoluted peaks was used for the calculation of crystallinity [27].

#### 2.2.4. Thermal Properties

Differential Scanning Calorimetry (DSC) was performed using a DSC Q2000 instrument (TA Instruments, New Castle, FL, USA). The samples were placed in hermetic aluminum pans and analyzed with a heating and cooling rate of 10 °C/min over a temperature range of −100 °C to 200 °C through three heating cycles. The melting temperatures ™ and melting enthalpies (ΔH_m_) were determined by analyzing the endotherm peak’s temperature and area, respectively, during the first heating cycle. The glass transition temperature (T_g_) was analyzed by endothermic slope observed during the last heating ramp. Thermogravimetric Analysis (TGA) was performed in a Thermogravimetric Analyzer Setaram Labsys EVO (Steram, Sophia Antipolis, France) with a weighing precision of +/−0.01%. Samples were placed in aluminum crucibles (8.6–16.3 mg) and analyzed in argon atmosphere with a temperature range between 25 °C and 500 °C at a rate of 10 °C/min. The degradation temperature (T_deg_) was considered the point where the sample had 5% mass loss (in the case of FucoPol, this weight loss was only considered after 150 °C due to water evaporation mass loss). The maximum degradation temperature was considered the value after major mass loss.

### 2.3. Preparation of PHBHVHHx Cast Films

Films were obtained by casting a biopolymer chloroform solution (20 mL, 9.5% *w*/*v*) into glass Petri dishes (with a diameter of 10 cm) and placing them in the fume hood inside a desiccator at room temperature until complete solvent evaporation. The films were kept at room temperature in a closed glass petri dish until use.

### 2.4. Preparation of PHBHVHHx Porous Scaffolds

#### 2.4.1. PHBHVHHx Scaffolds

For preparation of the porous scaffolds, 10 mL of the biopolymer solution in chloroform was mixed with 1 mL of deionized water and stirred with a magnetic stirrer until a stable emulsion formed (±1 h). Different biopolymer concentrations were tested, namely, 3.3%, 6.7%, and 9.5% (*w*/*v*). The resulting emulsions were transferred to 5 cm Petri dishes and left in the fume hood inside a desiccator at room temperature until complete solvent (water and chloroform) evaporation. The scaffolds were kept at room temperature in a closed glass petri dish until used.

#### 2.4.2. PHBHVHHx:FucoPol Scaffolds

The PHBVHHx:FucoPol scaffolds were obtained by emulsifying PHBHVHHx solutions with an aqueous FucoPol solution. PHBHVHHx was dissolved in chloroform at concentrations of 6.7 or 9.5% (*w*/*v*). FucoPol was dissolved in deionized water at concentrations of 0.1, 0.5, or 1.0% (*w*/*v*). An amount of 10 mL of the PHBHVHHx solution was mixed with 1 mL of the FucoPol solution, and the mixtures were stirred with a magnetic stirrer until an emulsion formed (±1 h) with no visible phase separation. The resulting emulsions were then transferred to 5 cm Petri dishes and left in the fume hood inside a desiccator at room temperature until complete solvent (water and chloroform) evaporation. The biopolymers were kept at room temperature in a closed glass petri dish until use.

### 2.5. Characterization of the Biopolymeric Structures

The developed PHBHVHHx cast films and the emulsion templated scaffolds (i.e., the PHBHVHHx:water and the PHBHVHHx:FucoPol scaffolds) were characterized in terms of their morphology, water uptake degree, and mechanical properties.

#### 2.5.1. Morphology

Macroscopic characteristics such as color, texture, and homogeneity were assessed by visual inspection. The thickness of the scaffolds was measured using a micrometer (Elcometer, Manchester, England). For the Scanning Electron Microscopy (SEM) analysis, the samples were frozen in liquid nitrogen and broken to obtain smaller pieces that were mounted for SEM observation using double-sided carbon tape and aluminum stubs and sputter coated with gold-palladium (60/40%) alloy (Q150T ES, Quorum Technologies, Ringmer, UK). The analysis was performed using a benchtop scanning electron microscope (TM3030 Plus, Hitachi, Tokyo, Japan) with an acceleration voltage of 15 kV. The obtained SEM images were processed by ImageJ.

#### 2.5.2. Mechanical Properties

The samples were cut into rectangular-shaped test pieces (~50 × 15 mm), and the tensile tests were performed using a texture analyzer (Food Technology Corporation, Wales, England), operated with a tensile rate of 100 mm/min until break, using a load cell of 50 N, under ambient conditions. The Young’s Modulus (E, MPa) was determined as the initial slope of the curve, the tensile strength (σ, MPa) was taken at the highest point of the curve just before break, and the elongation at break (ε, %) was determined as the ratio of the length of the test piece at rupture point by its initial length. Five replicas were made for each sample.

#### 2.5.3. Statistical Analysis

The statistical differences between the three attained structures (PHBHVHHx non-porous film attained by solvent casting, emulsion-templated PHBHVHHx-based scaffolds with water, and FucoPol) for each mechanical parameter (Young’s Modulus, tensile strength, and elongation at break) were performed through a one-way ANOVA analysis with Bonferroni’s multiple comparison test in GraphPad Prism 5 with a 0.05 significance threshold.

## 3. Results and Discussion

### 3.1. Biopolymer Characterization

#### 3.1.1. Composition

The PHBHVHHx biopolymer accumulated by the MMC cells was a scl-mcl-PHA composed of 55 wt% 3HB, 21 wt% 3HV, and 24 wt% 3HHx (Table 1). Similar monomer ratios (55–68 wt% 3HB, 9–17 wt% 3HV, and 15–36 wt% 3HHx) were reported for the terpolyester synthesized by a MMC from fruit waste [8,9]. Bhubalan (2010) reported a PHBHVHHx synthesized by the recombinant strain *Cupriavidus necator* P(3HB)-4 fed with crude palm kernel oil as the main substrate and valerate as a co-substrate that had a lower 3HHx content (2–7 wt%) and 3HB and 3HV contents of 66–82 wt% and 15–32 wt%, respectively [28]. The recombinant *Escherichia coli* LS5218 (fadR atoC [Con]) was also reported to synthesize PHBHVHHx with a monomer composition within the same ranges by cultivation on dodecanoic acid and different odd-carbon fatty acids [29]. Lower 3HV contents were reported for the biopolymers produced by *Rhodospirillum rubrum* ATCC 25,903 (6 wt%) [30], the recombinant *Aeromonas hydrophila* 4AK4 strains (1–13 wt%) [11,12,31,32], and the recombinant *C. necator* P(3HB)-4 (3–18 wt%) [10,28]. For those strains, the 3HHx content varied between 3 and 21 wt% (Table 1). The observed variability of PHBHVHHx monomers’ content is a result of the distinct microbial PHA-producing systems that were fed with different substrates, thus translating into biopolymers with different compositions.

FucoPol was composed of fucose (34.33 ± 2.05 %mol), glucose (33.12 ± 2.02 %mol), galactose (26.21 ± 2.04 %mol), and glucuronic acid (9.93 ± 1.06 %mol), which is the sugar composition characteristic of the polysaccharide FucoPol (30–36 %mol Fucose; 25–34 %mol Glucose; 22–29 %mol Galactose; 9–10 %mol glucuronic Acid) [33].

#### 3.1.2. Molecular Mass Distribution

The PHBHVHHx biopolymer had a M_w_ of 0.9 × 10^5^ Da (Table 1), a value that is among those reported for several mcl-PHAs (0.6 × 10^5^–4.1 × 10^5^ Da) [34] and scl-PHAs (1.2 × 10^5^–3.5 × 10^6^ Da) [35,36,37]. Higher values, between 3.0 × 10^5^ Da and 18.4 × 10^5^ Da, were reported for the PHBHVHHx synthesized by different bacteria, namely, the recombinant species of *Aeromonas hydrophila* 4AK4 and *Cupriavidus necator* P(3HB)-4 (Table 1). The biopolymer’s PDI was 2.2, which is within the average range reported for mcl-PHAs (1.60–4.40) [34], but higher than the average values (1.53–2.60) reported for different terpolyesters produced by the recombinant strains (Table 1). Such differences in molecular mass distribution among PHBHVHHx terpolyesters might be explained by numerous reasons, including the producing microorganisms, media composition and cultivation conditions, downstream processing, and even the cells’ stage of growth upon harvesting [34,38]. Additionally, the monomeric proportion in the biopolymer can also influence the resulting M_w_ and PDI [39].

On the other hand, FucoPol had an average M_w_ of 1.40 × 10^6^ Da, together with a PDI of 1.18, which are similar to previously reported values (1.7 × 10^6^ Da–5.8 × 10^6^ Da and 1.3–1.9, respectively) [40]. The high M_w_ observed in FucoPol gives this biopolymer the capacity to act as a thickening and emulsifying agent to form viscous solutions and stabilize emulsions [22]. Moreover, such properties, together with FucoPol’s inherent wound healing ability, render this biopolymer of great interest for the development of novel biomaterials.

**Table 1:** Composition, molecular mass distribution, and thermal properties of PHBHVHHx produced by different microbial sources (MMC, Mixed Microbial Culture; 3HB, 3-hydroxybutyrate; 3HV, 3-hydroxyvalerate; 3-HHx, 3-hydroxyhexanoate; M_w_, molecular weight; PDI, polydispersity index; T_g_, glass transition temperature; T_m_, melting temperature; T_deg_, degradation temperature; X_c_, crystallinity index; n.a., data not available; n.d., not detected).

Microbial Source	Composition (wt%)			M_w_ (×10^5^ Da)	PDI	T_g_ (°C)	T_m1_ (°C)	T_m2_ (°C)	T_deg_ (°C)	X_c_ (%)	References
	3HB	3HV	3HHx								
MMC	55	21	24	0.9	2.2	−3.8	144	159	275	26.2	This study
MMC	55	9	36	n.a.	n.a.	n.a.	n.a.	n.a.	n.a.	n.a.	[9]
MMC	68	17	15	n.a.	n.a.	0.2	111	173	266	22.6	[8]
*Rhodospirillum rubrum* ATCC 25903	89	6	5	n.a.	n.a.	n.a.	n.a.	n.a.	n.a.	n.a.	[30]
Recombinant *Aeromonas hydrophila* 4AK4	83	4	13	30.3	1.8	−1.3	113	n.a.	255.5	19.5	[12]
	83–91	1–7	5–15	3.0–8.0	1.5–2.2	−2.6 to −1.2	104–148	n.a.	249–273	14.2–22.7	[11]
	48–75	13–24	12–28	8.1–12.7	2.1–2.9	−1.9 to −12.5	54.2–101	n.a.	247–258	n.a.	[32]
Recombinant *Cupriavidus necator* P(3HB)-4	71–82	7–18	11	16.0–18.4	2.0–2.2	n.a.	141–143	n.a.	n.a.	14.5–27.5	[10]
	66–94	3–32	2–7	3.3–4.6	2.0–2.6	−4.7 to −0.8	91–129	139–148	n.a.	19.2–45.2	[28]
Recombinant *Cupriavidus necator* Re2133/pCB81	66 61 54	12 21 36	22 18 10	n.a. n.a. n.a.	n.a. n.a. n.a.	n.d. n.d. −2.8	70 133 152	n.a. n.a. n.a.	n.a. n.a. n.a.	13.4 5.4 7.2	[41]
Recombinant *E. coli* LS5218	61–91 69–79 58–79	0–33 0–22 0–31	4–13 8–21 9–21	n.a. n.a. n.a.	n.a. n.a. n.a.	n.a. n.a. n.a.	n.a. n.a. n.a.	n.a. n.a. n.a.	n.a. n.a. n.a.	n.a. n.a. n.a.	[29]

#### 3.1.3. X-ray Diffraction

**Figure 1:**
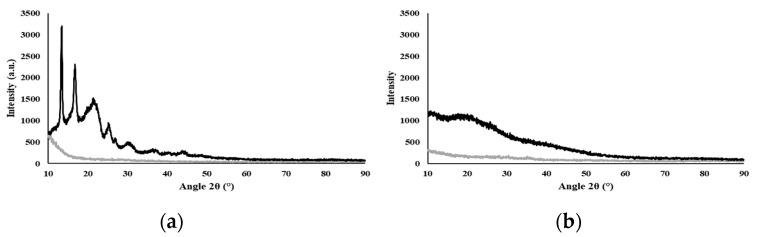
X-ray diffractogram of the biopolymers, where Std is the silicon sample holder and the samples are PHBHVHHx (**a**) and FucoPol (**b**).

The diffractogram (Figure 1a) shows the typical behavior of a semi-crystalline biopolymer, exhibiting peaks at 2θ equal to 13.08°, 16.36°, 21.53°, 25.05°, and 26.86°, which correspond to the (020), (110), (111), (130), and (040) lattice planes of the orthorhombic unit cell of PHB, as described by Melendéz-Rodriguéz (2018) [42]. This crystalline fraction of the biopolymer is imparted by its high content of 3HB monomers (55 wt%) [43]. The broad peak at 2θ around the 20° region relates to the amorphous fraction due to the incorporation of 3HV (21 wt%) and 3HHx (24 wt%) monomers, which significantly reduce the degree of crystallinity of the biopolymer [44]. It has been shown for P(3HB-co-3HHx) copolymers that increasing their content in the 3HHx monomer leads to lower crystallinity degree values [8]. The observed semi-crystallinity of PHBHVHHx can also be explained by the co-crystallization of 3HB and 3HV [10]. A similar behavior was reported for the PHBHVHHx produced by *A. hydrophila* 4AK4 with 3HB, 3HV, and 3HHx contents of 71–82 wt%, 7–18 wt%, and 11 wt%, respectively [10]. For the polysaccharide FucoPol, which was completely amorphous, no crystalline peaks were observed (Figure 1b), in accordance with previous reports [23].

#### 3.1.4. Thermal Properties

**Figure 2:**
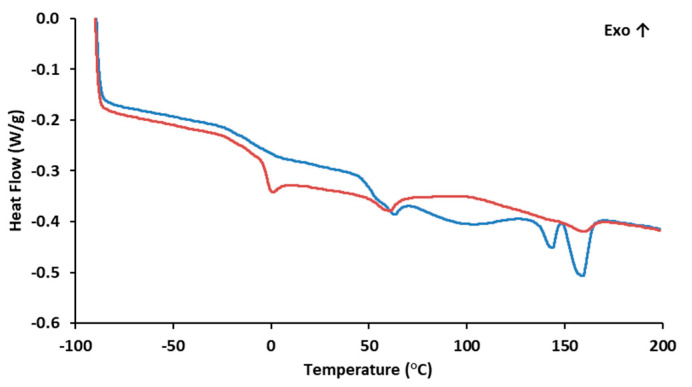
Differential Scanning Calorimetry of PHBHVHHx: 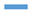 1st heating ramp; 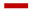 2nd heating ramp.

The DSC curve (Figure 2) for PHBHVHHx shows two melting endotherms at 144 °C (T_m1_) and at 159 °C (T_m2_) (Table 1). This phenomenon is observed in certain PHBHVHHx polyesters and is likely associated with the semicrystalline behavior of these biopolymers. This behavior aligns with the previously demonstrated X-ray diffraction results and can be attributed to the high concentrations of 3HV and 3HHx monomers present [8,44]. This crystallinity reduction has an impact on the polymers’ melting temperatures and enthalpies, usually unfolding in the presence of two endothermic peaks, the first for the lowest crystalline density fractions of the terpolymer (3HV and 3HHx), and the second for the most thermodynamically stable 3HB-rich fractions, as described by Meléndez-Rodriguéz (2021) [8]. The PHBHVHHx terpolymer described by Meléndez-Rodríguez (2021) had T_m1_ and T_m2_ values of 111 °C and 173 °C [8], respectively, which were higher than the melting temperatures determined in the present study. This fact may be related to the higher 3-HB content of the polymers reported by Meléndez-Rodriguéz (2021) [8]. On the other hand, the determined T_m_ values were higher than the ones reported for other terpolymers, where T_m1_ is usually between 91 and 129 °C and T_m2_ ranges from 139 to 148 °C (Table 1). Interesting enough, the T_m_ values attained in this work seem to be within the range found in the letter for PHBHV polymers (97–170 °C) [45]. Nevertheless, these values are all lower than those reported for the homopolymer PHB (173–180 °C) [46,47]. The lower melting temperatures seem to be related to the higher contents of 3HV and 3HHx in the biopolymer, since the incorporation of these monomers within 3HB domains apparently disturbs the possibility of the crystallization processes associated with 3HB, thus reducing the crystallinity of the terpolymers [8,11,48].

The biopolymer also exhibited some degree of melting transition, with a T_g_ of −3.8 °C (Figure 2), thus corroborating its semi-crystalline behavior as observed by the X-ray diffraction analysis. The low T_g_ value is associated with the biopolymer’s elastomeric behavior at room temperature [11]. The content of 3HV and 3HHx monomers seems to decrease the biopolymer T_g_ values. When compared with more crystalline PHB homopolymers that exhibit small transition events, usually within 0–5 °C [49,50,51], PHBHV and terpolyesters usually display T_g_ values ranging from −9 to 2 °C [45] and from −4.7 °C to −0.8 °C, respectively (Table 1). These differences in the T_g_ values are likely due to the presence of longer side chain monomers that decrease the biopolymer’s crystalline behavior [28].

**Figure 3:**
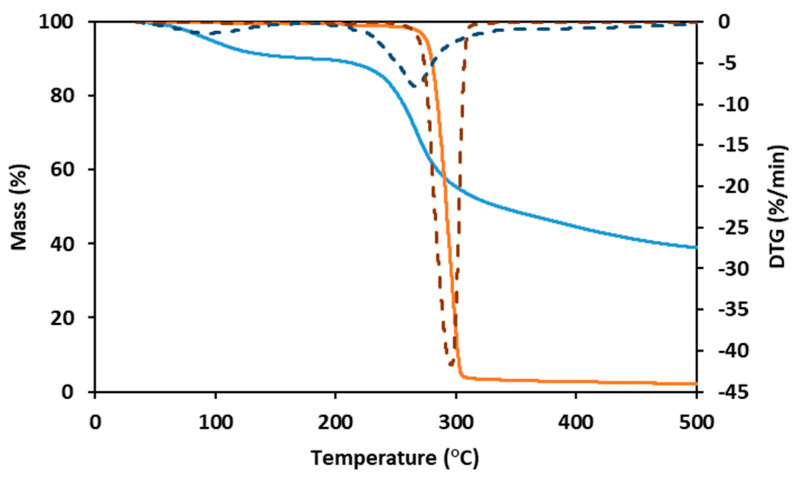
Thermogravimetric analysis (TGA) and derivative thermogravimetry (DTG) curves of PHBHVHHx (full orange line and dashed orange line, respectively) and FucoPol (full blue line and dashed blue line, respectively).

As shown by the TGA for the PHBHVHHx curve (Figure 3), the decomposition of the terpolyester is a fast, one-step process. The curve was stable until around 260 °C, suffering a weight loss of 5% at a temperature of around 275 °C and a major weight loss (92%) with a maximum degradation rate between 275 °C and 315 °C, with a char yield of approximately 2% at 500 °C (Figure 3). These results are within the ones reported for other terpolymers (247–273 °C) [8,11,32], somewhat higher than those reported for PHBHV (224–268 °C) [11,31,41] and PHBHHx copolymers (239–251 °C) [11], but slightly lower than for PHB homopolymers (290–300 °C) [6,11].

FucoPol displayed two weight loss steps in its TGA curve (Figure 3). The first occurs between 40 °C and 170 °C, where the sample loses around 10% of its mass, likely due to water loss due to the hygroscopic properties of FucoPol. Afterward, the sample maintains its mass stablility until around 200 °C, suffering a weight loss of 5% at around 240 °C. The maximum degradation rate (corresponding to the second and major step of mass loss) is between 240 °C and 300 °C, where the sample loses around 30% of its mass, losing another 20% until 500 °C, resulting in a char yield of 38% (Figure 3). A similar behavior was reported for FucoPol and for other polysaccharides [40].

### 3.2. Characterization of PHBHVHHx Cast Films

#### 3.2.1. Morphology

**Figure 4:**
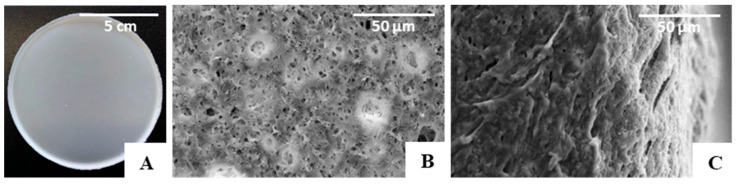
PHBHVHHx non-porous cast film’s macroscopic appearance. (**A**) SEM images of the surface, (**B**) cross-section, (**C**) amplified 1000×.

The PHBHVHHx films obtained by solution casting and solvent evaporation were white, opaque, and macroscopically homogeneous (Figure 4A), with a thickness of approximately 200 µm. As revealed by the SEM analysis, the films presented some irregularities on their surface with apparently some degree of porosity (Figure 4B), which, however, was not perceivable by the cross-section images (Figure 4C). Similar morphological features were reported for other PHBHVHHx-cast films [52,53].

#### 3.2.2. Mechanical Properties

The PHBHVHHx cast film had a Young’s modulus of 78.3 MPa, a tensile strength of 5.1 MPa, and an elongation at break of 269.2% (Table 2). These values are lower than those reported for PHB cast films, which usually have higher tensile strengths (18.4–40.1 MPa) and Young’s modulus values (1510–4600 MPa), together with low elongation at break values (1.6–4.5%) (Table 2). This is consistent with the more rigid and stiff nature of PHB films compared with co-polymers and terpolymers. The incorporation of 3HV or 3HHx monomers into the macromolecule renders PHBHV and PHBHHx co-polymers with lower Young’s modulus (45–2700 MPa) and tensile strength (4.1–21.8 MPa) values and higher elongation at break (8.8–113%), since these parameters depend on the 3HV and 3HHx contents in the polymeric chain (Table 2).

Higher Young’s modulus values were reported for the cast films prepared with the terpolymers produced by *A. hydrophila* 4AK4 (285–290 MPa) [11,12], which may be related to their lower contents in 3HV (3–5 wt%) and 3HHx (8–12 wt%) monomers (Table 2). As suggested by Zhao (2007) [11], increasing the polymer’s content in 3HV and 3HHx with the concomitant decrease of its 3HB content translates into a decrease in the biopolymer’s tensile strength. This is shown for the terpolymer produced by A. *hydrophila* 4AK4 from lauric acid and valerate, which was composed of a lower 3HB content (55 wt%) and higher 3HV and 3HHx contents (26 and 19 wt%, respectively). The resulting cast film had a lower Young’s modulus (2.0 MPa) together with a significantly lower tensile strength (0.3 MPa) [32]. These Young’s modulus values fall within those reported for soft tissues that range between 0.4 and 350 MPa [1]. Furthermore, according to Holzapfel (2001) [57], the skin’s tensile strength is typically within 1–20 MPa, a range that includes the value found for the MMC terpolymer (5.0 MPa). These findings support the use of PHBHVHHx in tissue engineering applications, with the added advantage of the possibility of tuning the scaffolds’ mechanical properties by adjusting the biopolymer’s monomer composition.

### 3.3. Preparation of the PHBHVHHx Emulsion-Templated Scaffolds

**Figure 5:**
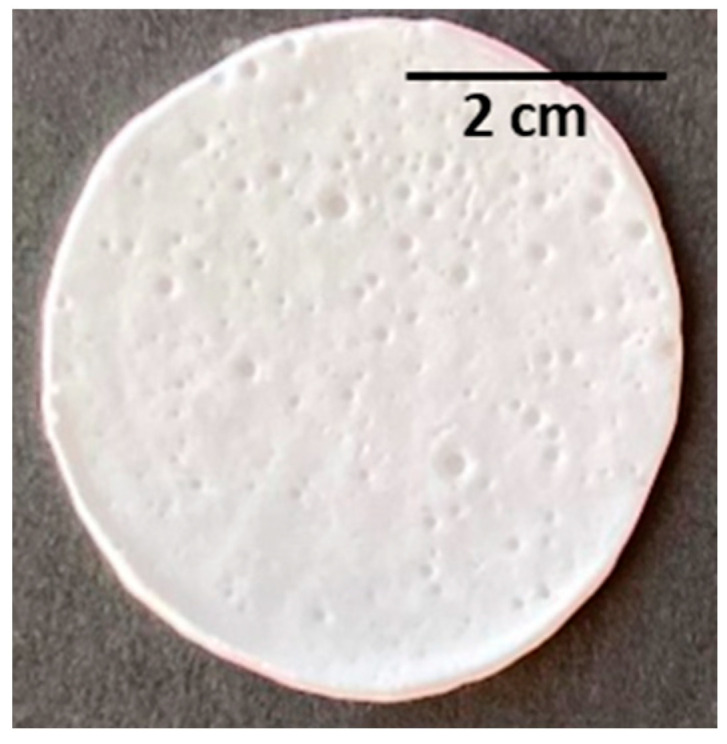
PHBHVHHx emulsion-templated scaffold obtained from the emulsification of deionized water and a polymer chloroform solution at 9.5% *w*/*v* (with a ratio of 1:10 *v*/*v*).

The porous scaffolds were prepared by the emulsion templating method, and one of the main challenges of this procedure was producing porous structures with interconnected pores [58]. Given these, it was tested at three different concentrations of PHBHVHHx solutions in chloroform (3.3, 6.7, or 9.5 wt%) as the continuous phase in order to find a suitable concentration for emulsion stabilization. The dispersed phase was composed of deionized water that was mixed with the polymer solutions at a ratio of 1:10 (*v*/*v*), resulting in white, stable, and homogeneous emulsions, similar to those reported by Esmail (2021) for emulsion-template PHB and PHBHV scaffolds [6]. The porous scaffolds were obtained upon casting the prepared emulsions, followed by solvent evaporation. The prepared scaffolds were white with an opaque appearance and an irregular surface. They had thickness values ranging from 160 to 300 µm and were flexible, similar to the cast films, as shown for the scaffold prepared with the 9.5% polymer solution (Figure 5). These macroscopic features were similar to those of PHB and PHBHV porous scaffolds prepared by the emulsion-templating strategy [6].

**Figure 6:**
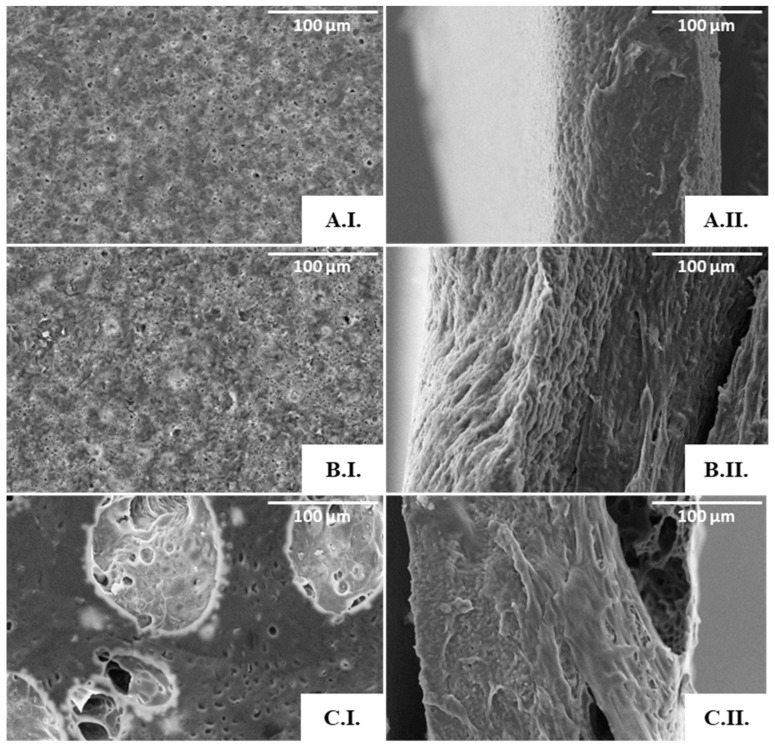
SEM images of the PHBHVHHx emulsion-templated scaffolds obtained from the 3.3% (**A**), 6.7% (**B**), and 9.5% (**C**) polymer solutions: surface (**I**) and cross-section (**II**) amplified 500×.

As shown by the SEM analysis (Figure 6), no porous structures were observed in the scaffolds obtained from the 3.3% (Figure 6(A.I.,A.II.)) or the 6.7% (Figure 6(B.I.,B.II.)) polymer solutions. An apparent porous surface, similar to that observed for the cast films, could be seen in these scaffolds (Figure 4), but their cross-section images revealed non-homogeneous and mostly compact structures (Figure 6(A.II.,B.II.)). These findings can be related to the instability of the emulsions during the solvent evaporation process, which resulted in scaffolds with a rather wide thickness range (from 160 to 300 µm).

On the other hand, the scaffolds prepared from the 9.5% polymer solution (Figure 6(C.I.)) comprised pores of different sizes, including macropores with a diameter of approximately 100 µm, dispersed throughout a microporous structure (Figure 6(C.I.)). Furthermore, the pores seemed to be interconnected through tunnel-like structures (Figure 6(C.II.)). Given these results, this scaffold was chosen for further characterization. This scaffold has a structure similar to that of PHB, PHBHV, and PHBHHx scaffolds attained by the solvent casting with particulate leaching (SCPL) strategy [5,59]. It also resembles the scaffolds of PHB, PHBHV, and PHBHV50PCL50 prepared with the emulsion templating technique [6,60]. Different polymeric structures were also reported to display such features, for instance, PLA [61], chitosan/gelatine [62], and polycaprolactone (PCL) scaffolds [63].

Aiming to improve the emulsions’ stability, FucoPol was tested as a bioemulsifier. For that, different concentrations of PHBHVHHx in the organic phase (6.7 and 9.5%, *w*/*v*) were tested. To determine which continuous phase concentration would result in a more stable emulsion, blends were prepared using 6.7 and 9.5 wt% of PHBHVHHx solutions with a FucoPol solution at 0.1 %*w*/*v*. After emulsification, the mixtures were kept at room temperature for 72 h. The emulsion with 6.7 wt% PHBHVHHx and FucoPol at 0.1 %*w*/*v* was the only one that showed no phase separation, being thus selected for further testing using two other FucoPol concentrations, 0.5 and 1.0% (*w*/*v*).

**Figure 7:**
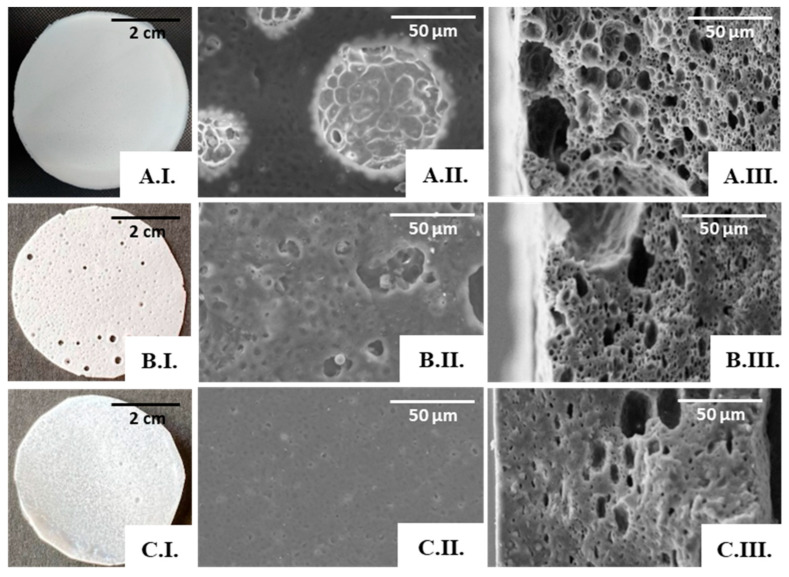
PHBHVHHx:FucoPol scaffolds obtained by emulsion-templating with PHBHVHHx solution at 6.7% and FucoPol solutions at concentrations of 0.1 wt% (**A**), 0.5 wt% (**B**), and 1.0 wt% (**C**). Macroscopic appearance (**I**); SEM images: surface (**II**) and cross-section (**III**), amplified 1000×.

Macroscopically, the PHBHVHHx:FucoPol scaffolds presented distinct characteristics (Figure 7). For the lowest FucoPol concentration tested (0.1 wt%), the surface was compact, homogeneous, white, and opaque (Figure 7(A.I.)), with the structures presenting a thickness of approximately 159 µm and a flexibility similar to that observed earlier for the scaffold prepared from the PHBHVHHx:water emulsion. On the other hand, the scaffolds prepared from the 0.5 wt% FucoPol aqueous solution were much thinner (117 µm) and presented macroscopically visible holes on the surface (Figure 7(B.I.)), thus evidencing their non-homogeneous nature. Further increasing the concentration of FucoPol to 1.0 wt% resulted in compact and homogeneous scaffolds with a thickness of 145 µm (Figure 7(C.I.)).

The SEM analysis also supported the macroscopic findings. The scaffold prepared from the 0.1 wt% FucoPol solution presented a porous surface (Figure 7(A.II.)), and pores were also observed in the cross-section images (Figure 7(A.III.)), with visible interconnected pores. However, the diameter of the pores was not homogeneous, decreasing in size throughout the thickness of the scaffold; these could be associated with the emulsion’s stability during the solvent evaporation process.

Increasing the FucoPol concentration in the dispersed phase led to an evident decrease in porosity and pore volume, as depicted in the cross-section images of the scaffolds prepared with 0.5 wt% (Figure 7(B.III.)) and with 1.0 wt% FucoPol solutions (Figure 7(C.III.)). The dispersed phase displayed increased viscosity concomitant with the higher FucoPol concentration in those solutions [22], which may have led to a coalescence of the internal phase of the emulsion for a concentration of 0.5 wt%, resulting in macroscopic holes (Figure 7(B.I.)) and larger pores on the scaffold’s surface (Figure 7(B.II.)), and an almost total loss of porosity on the surface for a concentration of 1 wt% (Figure 7(C.II.)).

The presence of FucoPol in the emulsions seems to enhance the scaffold’s porosity and pore interconnectivity when compared with the structures attained previously using the water emulsion method, provided the bioemulsifier concentration is kept low. Therefore, the porous scaffold prepared from a 0.1 wt% FucoPol solution was chosen for further characterization.

### 3.4. Mechanical Properties of the PHBHVHHx Emulsion-Templated Scaffolds

There was a significant change in the porous scaffolds’ mechanical properties when compared with those of the cast films. Although some statistical decrease of the Young’s Modulus was noticed, from 78.3 MPa for the cast films to 60 MPa for the water emulsion templated scaffold, the tensile strength was reduced significantly from 5.1 MPa to 3.6 MPa, and there was a steep statistical decrease of the elongation at break from 269.2 to 56% (Figure S1 and Tables S1–S3). The PHBHVHHx:FucoPol porous scaffolds, prepared from a 0.1% (*w*/*v*) FucoPol solution, had a stress-strain curve similar to that obtained for the water emulsion porous scaffold, displaying a Young’s Modulus of 85 MPa, which was slightly higher than the values found for the cast PHBHVHHx films (78.3 MPa) with no statistical significance and for the PHBHVHHx porous scaffold prepared by water emulsion (60 MPa) (Figure S1). These results might correlate with a decrease in the scaffold’s elasticity, as shown by its lower elongation at break (52%), and an increased tensile strength (4.4 MPa) when compared with the results observed for the porous scaffold prepared by water emulsion (56% and 3.6 MPa, respectively). However, for the elongation at break values, the differences between these two scaffolds were not significant and had low statistical relevance to the tensile strength (Figure S1). Nevertheless, this is still an interesting behavior considering that the PHBHVHHx:FucoPol porous scaffold was thinner (159 µm) and had a higher porosity, as shown by the SEM micrographs (Figure 7 (A.III.)). Moreover, the addition of FucoPol to PHBHVHHx scaffolds seems to enhance the materials’ tensile strength.

The scaffolds’ mechanical strength is a fundamental property for cell culture since they must support cell proliferation and cell mobility [64]. Considering the tensile strength reported for native human dermis (1.03 to 3.10 MPa) [65], the slightly higher value obtained for the emulsion-template scaffolds of PHBHVHHx (3.6 MPa and 4.4 MPa) might be able to serve as a support for cell activities without suffering significant contraction of its volume. Moreover, the Young’s Modulus of the developed structures is within the values reported for soft tissue applications (0.4–350 MPa) [1]. When compared with other porous structures reported in the literature prepared with the same technique (Table 3), the Young’s Modulus obtained in this study (60 and 85 MPa) was considerably higher than those of fibrin, silk fibroin, and collagen/fibrin porous scaffolds (0.23–2 MPa) [66], thus supporting the advantage of the developed PHBHVHHx scaffolds. Moreover, the presence of FucoPol in PHBHVHHx structures could enhance the scaffold’s wound healing ability, making it more suitable for tissue engineering applications.

## 4. Conclusions

This study demonstrated the valuable properties of the porous scaffolds based on the terpolyester PHBHVHHx prepared by emulsion-templating with an aqueous solution of the bioactive polysaccharide FucoPol. This technique allowed for the combination of the two biocompatible and biodegradable natural polymers, resulting in 3D porous structures with enhanced physical and chemical properties. FucoPol acted not only as a bioemulsifier for emulsion stabilization, improving scaffold production by the emulsion templating technique, but it also enhanced the structural features of PHBHVHHx when compared with cast films. Moreover, incorporating FucoPol’s bioactivity into the PHBHVHHx scaffolds could be an added advantage for their use in tissue engineering. To assess this, cell adhesion and proliferation tests are needed to ensure the scaffold’s applicability in the biomedical area. It could also be interesting to study different methods to obtain scaffolds that combine PHBHVHHx and FucoPol to estimate the best procedures to attain a structured material that gathers the main advantages of both biopolymers.

## Figures and Tables

**Table 2:** Mechanical properties of PHA films with different monomeric compositions prepared by solution casting and solvent evaporation (E—Young’s Modulus; σ—Tensile strength; ε—Elongation at break).

Polymer (Monomers, wt%)	E (MPa)	σ (MPa)	ε (%)	References
PHBHVHHx (55:21:24)	78.3 ± 6.9	5.1 ± 0.2	269.2 ± 52.1	This study
PHBHVHHx (64:18:18)	750	12.5	4.25	[8]
PHBHVHHx (89:3:8)	285	5.0	264	[12]
PHBHVHHx (83:5:12)	290	15.7	340	[11]
PHBHVHHx (55:26:19)	2.0	0.3	133	[32]
PHBHHx (88:12)	130–498	4.1–9.4	104–113	[11,12,32]
PHBHV (97:3)	2700	21.8	12.1	[54]
PHBHV (85:15)	531	10.9	8.8	[55]
PHBHV (65:35)	45	1.22	12.9	[55]
PHBHV (87:13)	4000	35	2	[8]
PHB	1510–4600	18.4–40.1	1.6–8.2	[11,12,32,54,56]

**Table 3:** Mechanical properties for the developed emulsion-templated PHBHVHHx-based scaffolds and for emulsion-templated scaffolds reported in the literature (E—Young Modulus; σ—Tensile strength; ε—Elongation at break).

Polymer (Monomers, wt%)	Method	Thickness (mm)	E (MPa)	σ (MPa)	ε (%)	References
PHBHVHHx (55:21:24)	Water emulsion	241	60	3.6	56	This study
	FucoPol (0.1 wt%) emulsion	159	85	4.4	52	This study
PHBHV (75:25)	Water emulsion	260	0.11	3.4	14.8	[6]
PHBHV (80:20)	Electrospinning	60	434–1166	7.1–18.9	2.6–2.9	[42]
PHB	Water emulsion	610	0.07	3.18	13.6	[6]
	Particle Leaching	45	1815	13.5	0.9	[57]
Collagen	oil-in-water emulsion	n.a.	1–2	7.8–9.7	n.a.	[66]
Collagen/Fibrin	oil-in-water emulsion	n.a.	1–3	12.0–16.0	n.a.	[66]
Fibrin	oil-in-water emulsion	n.a.	1–2	4.0–5.1	n.a.	[66]

## Data Availability

The data presented in this study are available on request from the corresponding author.

## Supplementary Materials

The following supporting information can be downloaded at: https://www.mdpi.com/article/10.3390/polym15132945/s1, Figure S1: Mechanical properties of PHBHVHHx non-porous film attained by solvent casting, emulsion-templated PHBHVHHx-based scaffolds with water and FucoPol; Table S1: One-way ANOVA results for the Tension at break parameter with Bonferroni’s Multiple Comparison Test for the produced structures (PHBHVHHx non-porous film attained by solvent casting, emulsion-templated PHBHVHHx-based scaffolds with water and FucoPol); Table S2: One-way ANOVA results for the deformation at break parameter with Bonferroni’s Multiple Comparison Test for the produced structures (PHBHVHHx non-porous film attained by solvent casting, emulsion-templated PHBHVHHx-based scaffolds with water and FucoPol); Table S3: One-way ANOVA results for the Young Modulous parameter with Bonferroni’s Multiple Comparison Test for the produced structures (PHBHVHHx non-porous film attained by solvent casting, emulsion-templated PHBHVHHx-based scaffolds with water and FucoPol).
