# Supplementary material for: Preparation of Porous Scaffold Based on Poly(3-hydroxybutyrate-co-3-hydroxyvalerate-co-3-hydroxyhexanoate) and FucoPol
Source: Polymers (Basel). 2023 Jul 4;15(13):2945. doi: 10.3390/polym15132945 (PMC10347064; doi:10.3390/polym15132945)
Supplement: Supplementary file 1 [file polymers-15-02945-s001.zip › polymers-2454314-supplementary.pdf]

Supporting Information

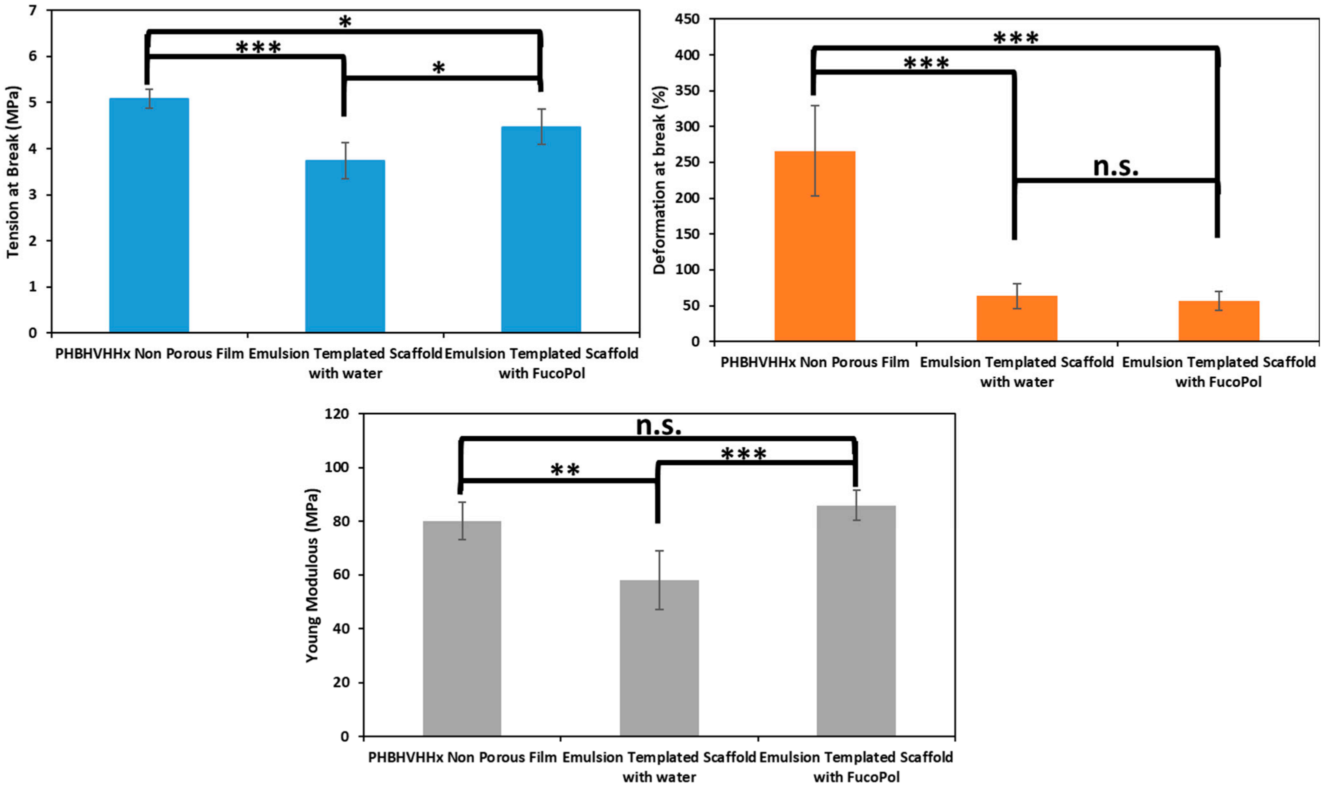

Figure S1 – Mechanical properties of PHBHVHHx non-porous film attained by solvent casting, emulsion-templated PHBHVHHx-based scaffolds with water and FucoPol. ■ - Tension at break results (MPa); ■ - Deformation at break results (%); ■ - Young Modulous results (%). Results were expressed using the mean ± standard deviation of 5 samples of each structure (film and scaffolds). Statistically significant differences comparing each sample were calculated with one-way ANOVA with Bonferroni's multiple comparison test (*p≤0.05, **p≤0.01, ***p≤0.001, n.s. – not significant).

Table S1 – One-way ANOVA results for the Tension at break parameter with Bonferroni's Multiple Comparison Test for the produced structures (PHBHVHHx non-porous film attained by solvent casting, emulsion-templated PHBHVHHx-based scaffolds with water and FucoPol).

**Tension at Break (MPa)**					
**Parameter**					
One-way analysis of variance					
P value	0,0001				
P value summary	***				
Are means signif. different? (P < 0.05)	Yes				
Number of groups	3				
F	20,09				
R squared	0,7700				
ANOVA Table	***SS***	***df***	***MS***		
Treatment (between columns)	4,551	2	2,275		
Residual (within columns)	1,359	12	0,1133		
Total	5,910	14			
Bonferroni's Multiple Comparison Test	***Mean Diff.***	***t***	***Significant? P < 0.05?***	***Summary***	***95% CI of diff***
PHBHVHHx non-porous film **vs** emulsion-templated scaffold PHBHVHHx-water	1,347	6,331	Yes	***	0.7558 to 1.939
PHBHVHHx non-porous film **vs** emulsion-templated scaffold PHBHVHHx-FucoPol	0,6134	2,882	Yes	*	0.02182 to 1.205
emulsion-templated scaffold PHBHVHHx-water **vs** emulsion-templated scaffold PHBHVHHx-FucoPol	-0,7340	3,449	Yes	*	-1.326 to -0.1424

Table S2 – One-way ANOVA results for the deformation at break parameter with Bonferroni's Multiple Comparison Test for the produced structures (PHBHVHHx non-porous film attained by solvent casting, emulsion-templated PHBHVHHx-based scaffolds with water and FucoPol).

**Deformation at break (%)**					
**Parameter**					
One-way analysis of variance					
P value	< 0.0001				
P value summary	***				
Are means signif. different? (P < 0.05)	Yes				
Number of groups	3				
F	47,40				
R squared	0,8876				
ANOVA Table	***SS***	***df***	***MS***		
Treatment (between columns)	141100	2	70530		
Residual (within columns)	17860	12	1488		
Total	158900	14			
Bonferroni's Multiple Comparison Test	***Mean Diff.***	***t***	***Significant? P < 0.05?***	***Summary***	***95% CI of diff***
PHBHVHHx non-porous film **vs** emulsion-templated scaffold PHBHVHHx-water	202,0	8,278	Yes	***	134.2 to 269.8
PHBHVHHx non-porous film **vs** emulsion-templated scaffold PHBHVHHx-FucoPol	209,3	8,577	Yes	***	141.5 to 277.1

emulsion-templated scaffold PHBHVHHx-water **vs** emulsion-templated scaffold PHBHVHHx-FucoPol	7,304	0,2994	No	ns	-60.51 to 75.12

Table S3 – One-way ANOVA results for the Young Modulous parameter with Bonferroni's Multiple Comparison Test for the produced structures (PHBHVHHx non-porous film attained by solvent casting, emulsion-templated PHBHVHHx-based scaffolds with water and FucoPol).

**Young modulous (MPa)**					
**Parameter**					
One-way analysis of variance					
P value	0,0004				
P value summary	***				
Are means signif. different? (P < 0.05)	Yes				
Number of groups	3				
F	16,30				
R squared	0,7309				
ANOVA Table	***SS***	***df***	***MS***		
Treatment (between columns)	2173	2	1087		
Residual (within columns)	800,0	12	66,67		
Total	2973	14			
Bonferroni's Multiple Comparison Test	***Mean Diff.***	***t***	***Significant? P < 0.05?***	***Summary***	***95% CI of diff***
PHBHVHHx non-porous film **vs** emulsion-templated scaffold PHBHVHHx-water	22,00	4,260	Yes	**	7.647 to 36.35
PHBHVHHx non-porous film **vs** emulsion-templated scaffold PHBHVHHx-FucoPol	-6,000	1,162	No	ns	-20.35 to 8.353
emulsion-templated scaffold PHBHVHHx-water **vs** emulsion-templated scaffold PHBHVHHx-FucoPol	-28,00	5,422	Yes	***	-42.35 to -13.65
